# Supplementary material for: In Vitro Expression Analysis Reveals HML6-c14 to Be an Attractive Research Target
Source: Biomolecules. 2023 Sep 12;13(9):1378. doi: 10.3390/biom13091378 (PMC10526471; doi:10.3390/biom13091378)
Supplement: Supplementary file 1 [file biomolecules-13-01378-s001.zip › biomolecules-2557581-supp-update.pdf]

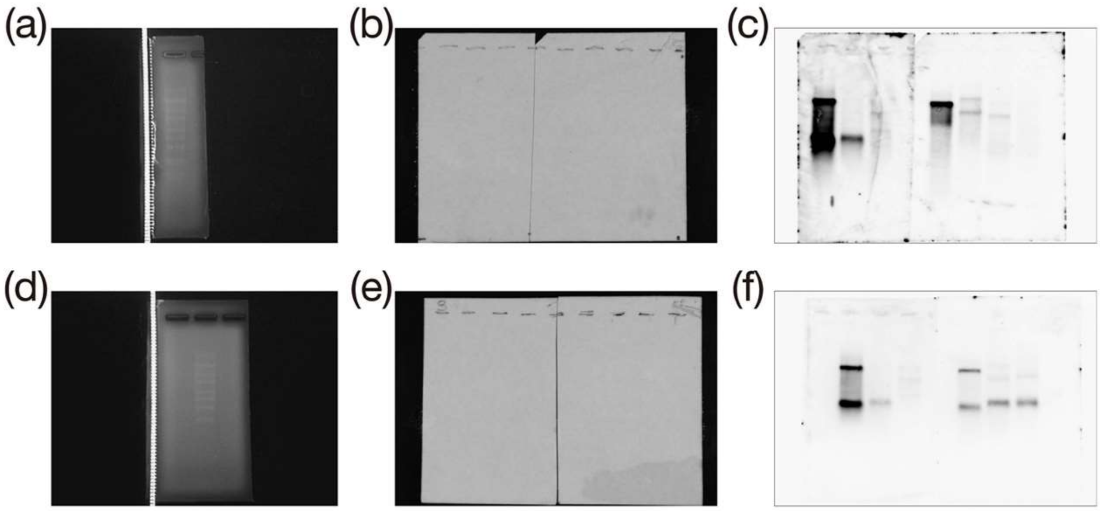

Figure S1. The original data used in Northern analysis in Figure 1c. (**a**) Data for probe B; the image of the stained gel containing the size marker lane (second from the right in Figure 1c). (**b**) Data for probe B; blots used for Northern with the well locations marked. The visible light image from the ImageQuant™ LAS 4000mini chemiluminescence imaging system. A blot cut in two can be observed. Three samples were electrophoresed on each blot. Although not used in the Figure 1c itself, it was used to confirm the position of markers (panel (**a**)) and probe signals (panel (**c**)). (**c**) Data for probe B; the image in the first lane from the left on the right sided blot was used as the result for probe B in Figure 1c. (**d**) Data for probe A and C; the image of the stained gel containing the size marker lane (leftmost lane in Figure 1c). (**e**) Data for probe A and C; blots used for Northern with the well locations marked. The method of acquiring the image is the same as that in panel (b). A blot cut in two can be observed. Three samples were electrophoresed on each blot. Although not used in the Figure 1c itself, it was used to confirm the position of markers (panel (**d**)) and probe signals (panel (**f**)). (**f**) Data for probe A and C; in both right and left blots, the images of the first sample from the left were used as the results for probe A and C in Figure 1c, respectively.

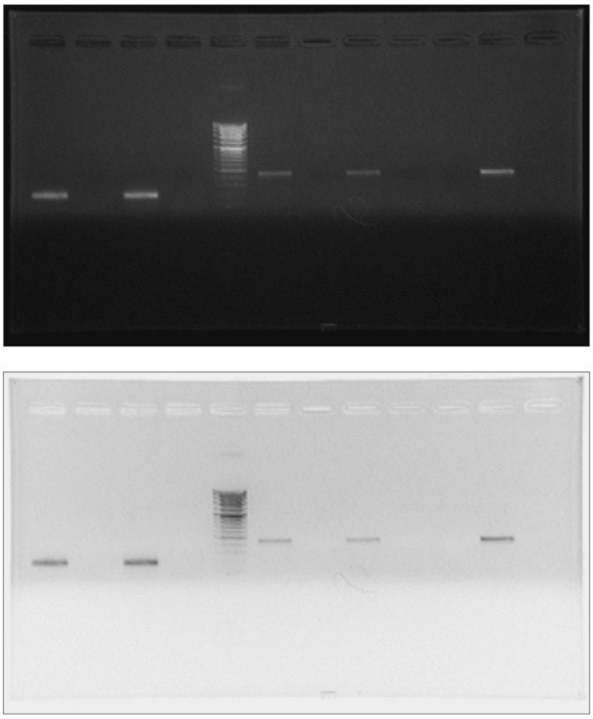

Figure S2. The original data used in RT-PCR in Figure 1d. (top) Image obtained by agarose gel electrophoresis after RT-PCR reaction and observed under ultraviolet (UV) light after staining with ethidium bromide The

fifth to eleventh lanes from the left were used in the upper panel of Figure 1d. (bottom) A negative image of the same file. This image was used for the actual Figure 1d.

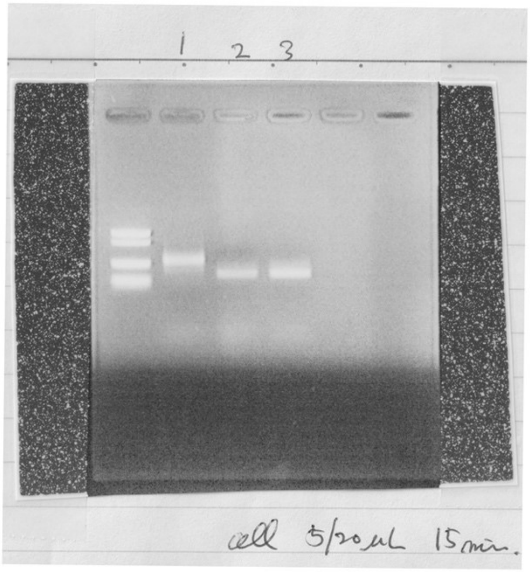

Figure S3. The original data used in 5′RACE experiment in Figure 2a. The first and second lanes from the left were used as size marker and the sample result in Figure 2a, respectively. This is a scanned image of a photo pasted into the author's research notes. Unfortunately, the digital image data from the CCD camera was lost.

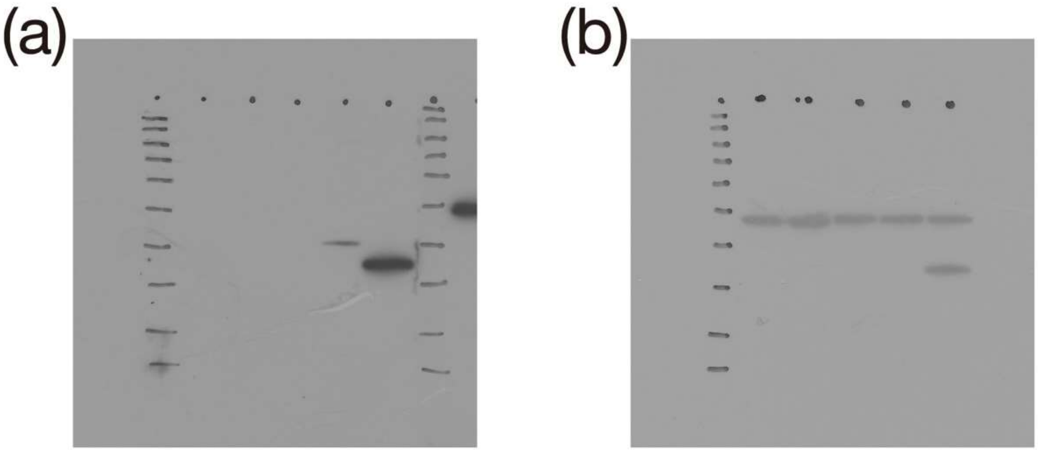

Figure S4. The original data used in Protein translation ability analysis in Figure 4c. (**a**) Fusion protein detection results using anti-FLAG antibody. The result of this blot was used in the top panel of Figure 4c. Because the two blots were detected side by side at the same time, the position of the marker of the blot on the right and the detected band on the 1st lane can be seen at the right edge of this figure. (**b**) Results for beta-actin used in the bottom panel of Figure 4c. The lower band in the rightmost lane is due to antibodies that were not completely stripped from GFP+FLAG fusion protein.

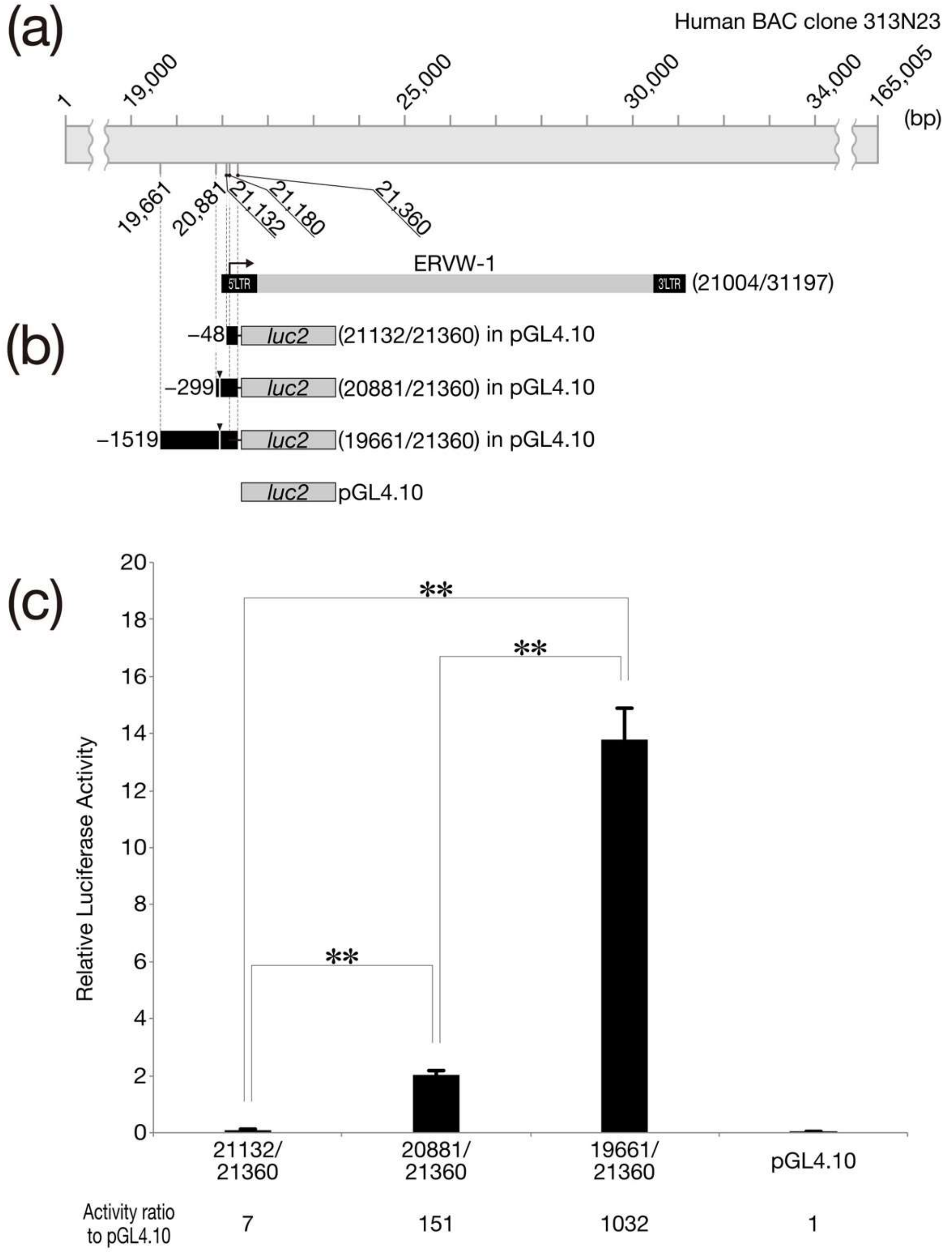

Figure S5. Promoter analysis of ERVW-1 performed to confirm the sensitivity of the DLR assay. (**a**) Schematic representation of human BAC Clone 313N23 compared to the ERVW-1 element. The numbers above and below the schematic of Clone 313N23 denote the positions of the numbered nucleotides. The arrow above the 5′-LTR indicates the location of the transcription start site[22]. (**b**) Schematic representation of the promoter constructs used in this study. The genomic fragment inserted in pGL4.10 is indicated on the left by a horizontal filled bar, denoting its location in BAC Clone 313N23 as depicted in Panel (**a**). The numbers in parentheses on the right indicate the ranges of the genomic fragments in BAC Clone 313N23. (**c**) Promoter analysis for the ERVW-1 gene. BeWo cells were transfected with 0.5 μg of the mixture of the indicated promoter construct and pGL4.74. Values are presented as mean and standard deviation (SD) from two independent transfection experiments, each performed in triplicate. Asterisks denote statistically significant differences (**, $p < 0.0001$) between indicated expression plasmid-transfected groups (two-tailed non-paired Student's *t*-tests). The numbers below the graph indicate the fold difference in expression following normalization to that obtained with the negative control (pGL4.10).
